# Simultaneous Establishment of Autologous Colorectal Cancer and Mesothelial Stromal Cell Lines from Malignant Ascites Reveals a Mesothelial‐Stromal FGFR3 Axis as a Potential Vulnerability in Peritoneal Metastasis

**DOI:** 10.1002/cam4.71804

**Published:** 2026-04-24

**Authors:** Yasuhiro Fukui, Hiroaki Kasashima, Zizhou Wang, Iguru Omori, Yukina Kusunoki, Kanae Echizen, Yoshiki Nonaka, Yuki Seki, Kenji Kuroda, Yuichiro Miki, Mami Yoshii, Tatsunari Fukuoka, Tatsuro Tamura, Masatsune Shibutani, Takahiro Toyokawa, Yu Muta, Yuki Nakanishi, Masakazu Yashiro, Naoko Ohtani, Kiyoshi Maeda

**Affiliations:** ^1^ Department of Gastroenterological Surgery Osaka Metropolitan University, Graduate School of Medicine Osaka Japan; ^2^ Department of Pathophysiology Osaka Metropolitan University, Graduate School of Medicine Osaka Japan; ^3^ Department of Gastroenterology and Hepatology Kyoto University, Graduate School of Medicine Kyoto Japan; ^4^ Molecular Oncology and Therapeutics Osaka Metropolitan University, Graduate School of Medicine Osaka Japan

**Keywords:** cell line establishment, colorectal cancer, mesothelial cell, peritoneal dissemination, tumor‐stroma interaction

## Abstract

Colorectal cancer (CRC) with peritoneal dissemination remains a major therapeutic challenge because of poor prognosis and limited treatment options. Experimental models that accurately recapitulate tumor–mesothelial interactions are scarce. Here, we report the establishment of a novel autologous paired model comprising a CRC cell line (OMUCR‐1) and matched cancer‐associated mesothelial cells (CAmeso), both simultaneously derived from the malignant ascites of the same patient. Lineage marker analysis using qPCR demonstrated that OMUCR‐1 selectively expressed epithelial markers (*EPCAM, KRT20*), whereas CAmeso strongly expressed mesothelial‐mesenchymal markers (ACTA2, MSLN) and lacked epithelial marker expression. These mutually exclusive expression patterns confirm that the two cell populations are phenotypically distinct and rule out cross‐contamination. OMUCR‐1 displayed strong tumorigenic capacity across multiple transplantation models. CAmeso enhanced CRC cell migration and invasion in vitro, and co‐transplantation with OMUCR‐1 resulted in larger tumors enriched with αSMA‐positive stromal components. RNA sequencing of co‐injected xenografts revealed increased expression of murine stromal Fgfr3. Treatment with the FGFR inhibitor BGJ398 reduced tumor growth and decreased stromal FGFR3‐positive components, suggesting that stromal FGFR3 may represent a potential microenvironmental vulnerability in CRC with peritoneal dissemination. This autologous CRC‐mesothelial system provides a physiologically relevant platform for dissecting tumor‐stroma interactions in peritoneal metastasis and may advance stromal‐targeted therapeutic strategies.

**Abbreviations**
ACTA2: actin alpha 2
CAmeso: cancer‐associated mesothelial cells
CM: conditioned medium
CRC: colorectal cancer
CT: computed tomography
CXCL12: C‐X‐C motif chemokine ligand 12
DEG: differentially expressed genes
EPCAM: epithelial cell adhesion molecule
FGFR: fibroblast growth factor receptor
IHC: immunohistochemistry
KRAS: Kirsten rat sarcoma viral oncogene homolog
KRT20: keratin 20
MMP: matrix metalloproteinase
MSLN: mesothelin
Nmeso: normal mesothelial cells
RT‐PCR: reverse transcription‐polymerase chain reaction
STR: short tandem repeat
TGF‐β: transforming growth factor β
TP53: tumor protein p53
αSMA: α‐smooth muscle actin

## 1 Introduction

Colorectal cancer (CRC) is a prevalent malignancy and the leading cause of cancer‐related mortality worldwide [1]. Despite advancements in surgical techniques, chemotherapy, and targeted therapies, the prognosis of patients with peritoneal dissemination remains poor, owing to limited treatment options and an incomplete understanding of tumor biology [2, 3]. A critical barrier to improving patient outcomes is the scarcity of experimental models that replicate the complex interactions between cancer cells and the peritoneal microenvironment [4].

Although CRC encompasses both colon and rectal tumors, these tumors differ in anatomical location, vascular drainage, local recurrence patterns, and interactions with surrounding tissues [5, 6, 7]. Rectal cancer exhibits distinct biological and clinical characteristics because of its location within the pelvis and its proximity to the peritoneal reflection. These differences may influence metastatic spread patterns and tumor‐peritoneal interactions. Most experimental CRC models do not define tumor origin, and models specifically derived from rectal cancer‐associated malignant ascites are extremely limited. Therefore, the establishment of a rectal cancer‐derived experimental platform is of particular importance for understanding peritoneal dissemination in this subset of CRC.

Fibroblast growth factor receptor (FGFR) signaling—particularly through FGFR3—has been implicated in tumor progression and stromal remodeling in several malignancies. For example, FGFR3 overexpression in the tumor microenvironment promotes cancer cell proliferation and invasion in gastric and bladder cancer [8]. However, stromal FGFR3 signaling's contribution to the peritoneal dissemination of CRC remains poorly understood. A physiologically relevant model incorporating patient‐derived stromal components may help clarify these mechanisms and identify novel therapeutic targets.

Mesothelial cells originating from the embryonic mesoderm are simple, non‐adhesive linings that cover serosal cavities and internal organs [9]. The cells are biologically active and capable of reactivating developmental programs under pathological conditions, including tissue injury, fibrosis, and inflammatory states. Recent studies demonstrate that tissue‐specific peritoneal fibroblasts promote CRC dissemination and outgrowth within the peritoneal cavity [10]. In peritoneal dissemination, metastatic tumor cells interact with the mesothelial cell layer. The peritoneal cavity is not merely a passive site of tumor implantation, but rather an active stromal niche supporting metastatic colonization [11]. Tumor‐ and immune‐derived inflammatory mediators, along with ascitic fluid accumulation, remodel the peritoneal microenvironment and increase its susceptibility to metastatic seeding [12]. Inflammation‐driven mesenchymal transition of mesothelial cells enhances their interaction with tumor cells, thereby facilitating both early implantation and the expansion of secondary lesions [13].

In this study, we aimed to highlight a significant advancement in CRC research: the simultaneous establishment of a novel rectal cancer cell line, OMUCR‐1, and cancer‐associated mesothelial cells (CAmeso) derived from the malignant ascites of a single patient with advanced rectal cancer. This dual derivation from the same clinical specimen offers a unique and physiologically relevant model for investigating tumor‐stroma interactions within the peritoneal cavity. Normal mesothelial cells (Nmeso) served as the control, obtained from the ascites of a patient with CRC without peritoneal dissemination, thereby enabling robust comparative analyses.

Notably, this is the first study documenting the concurrent derivation of rectal tumor and mesothelial cells from the same patient, enabling a novel autologous in vitro and in vivo model. This innovative approach facilitates the direct investigation of the dynamic crosstalk between cancer and mesothelial cells under conditions that closely mimic the in vivo tumor microenvironment. By leveraging this unique model, elucidate the mechanisms underlying peritoneal metastasis while identifying novel therapeutic targets that disrupt tumor‐stroma interactions in CRC.

The biological and molecular features of OMUCR‐1 cells, including their growth kinetics, tumorigenicity, and drug sensitivity, were comprehensively characterized in this study. The tumor‐promoting effects of CAmeso on CRC cell invasiveness and progression were also examined. Collectively, our findings provide a valuable platform for advancing studies into peritoneal dissemination and for developing more effective treatment strategies for patients with aggressive CRC.

## 2 Materials and Methods

### 2.1 Establishment of Cell Lines and Cell Culture

OMUCR‐1 was isolated from the malignant ascites of a male patient in his 40s diagnosed with peritoneal dissemination of CRC. The ascitic fluid collected was centrifuged, and the cell pellet was suspended in 10 mL of Dulbecco's modified Eagle's medium (DMEM; Fujifilm Wako Pure Chemical Corporation) supplemented with fetal bovine serum (FBS; Nichirei Bioscience Inc.), 0.5 mM sodium pyruvate (Sigma), and penicillin–streptomycin solution (Fujifilm Wako Pure Chemical Corporation). Floating cells were collected, resuspended in the medium, and labeled OMUCR‐1. Adherent mesothelial cells were immortalized by transfection with SV40 (Addgene; plasmid #1778) and hTERT (Addgene; plasmid #1773) genes. This allowed the simultaneously establishment of a cancer cell line (OMUCR‐1), and mesothelial cells (CAmeso) derived from the same host. In addition, mesothelial cells were derived from the ascites of another patient diagnosed with CRC without dissemination, serving as a normal control (Nmeso). Human colorectal tumor, line 116 (HCT116) cells were obtained from the American Type Culture Collection. After HCT116 cells were authenticated by STR profiling before distribution to eliminate contamination, we cryopreserved them up to the 5th passage. Cell lines were used in 3 months after thawing. All cell lines were regularly screened for mycoplasma contamination and were confirmed to be free of mycoplasma prior to experimentation. This study was approved by the Osaka Metropolitan University Ethics Committee, and written informed consent was obtained from all patients.

### 2.2 Growth Kinetics

Suspensions of 1.0 × 10^4^ OMUCR‐1 cells were incubated in 24‐well dishes with 1 mL DMEM containing 10% FBS. Cancer cells were counted daily using an EVE automatic cell counter (NanoEnTek). Doubling times were determined from the growth curve.

### 2.3 Growth Inhibition Assay

Three cytotoxic anticancer agents (5‐fluorouracil [5‐FU], oxaliplatin, and irinotecan) and two FGFR inhibitors (AZD4547 and BGJ398) were tested. Cell viability was assessed using the Cell Counting Kit‐8 (CCK‐8; Dojindo) according to the manufacturer's instructions. Cells were seeded in 96‐well plates (5 × 10^3^ cells per well) and treated with the indicated concentrations of drugs for 72 h at 37°C in a humidified atmosphere containing 5% carbon dioxide (CO₂). Subsequently, 10 μL of CCK‐8 solution was added to each well and incubated at 37°C for 1 h, and using a microplate reader, absorbance was measured at 450 nm.

### 2.4 Short Tandem Repeat Analysis (STR)

STR profiling was performed using the services of the Japanese Collection of Research Bioresources (JCRB) Cell Bank (Osaka, Japan) to ensure that no cross‐contamination occurred in the cell lines [14].

### 2.5 Next‐Generation Sequencing Analysis

Genomic DNA was extracted from OMUCR‐1 cells using NucleoSpin Tissue (MACHEREY‐NAGEL, 740952.10). Genomic mutations were detected using the Axen Cancer Panel 1 (Macrogen, Japan).

### 2.6 Tumorigenicity

Tumorigenicity assays were performed on OMUCR‐1 cells. OMUCR‐1 cells were transplanted into the subcutaneous tissue, rectum, and the intraperitoneal cavity of nude mice (Oriental Kobo) to evaluate their tumorigenic potential. Cell suspensions containing 1 × 10^6^ OMUCR‐1 cells were inoculated, and the tumor incidence was determined after > 8 weeks. Orthotopic rectal transplantation was performed as described in previous reports [15, 16]. Tumor specimens were washed in phosphate‐buffered saline (PBS) and fixed in 10% formalin for paraffin embedding and sectioning. All animal experiments were performed in compliance with the Osaka Metropolitan University Ethics Committee guidelines.

### 2.7 Reverse Transcription‐Polymerase Chain Reaction (RT‐PCR)

Quantitative real‐time RT‐PCR was performed as follows: total RNA was extracted from OMUCR‐1 and CAmeso cells using TRIzol (Life Technologies) and the RNeasy Plus Mini Kit (QIAGEN). cDNA was synthesized from 600 ng of RNA using the ReverTra Ace quantitative PCR (qPCR) RT Master Mix (TOYOBO). qPCR was conducted using THUNDERBIRD SYBR qPCR Mix (TOYOBO) on a QuantStudio real‐time PCR system (Thermo Fisher Scientific) under the following cycling conditions: 95°C for 10 min, followed by 40 cycles of 95°C for 15 s, and 60°C for 1 min. Relative mRNA expression levels were calculated using the ΔΔCt method, with glyceraldehyde‐3‐phosphate dehydrogenase as the internal control gene. All reactions were performed in triplicate. Semiquantitative RT‐PCR was performed as previously reported [17, 18]. Primer sequences used in this study are summarized in Table S1.

### 2.8 Immunohistochemistry (IHC)

Immunohistochemical staining was conducted using the following antibodies: anti‐α‐smooth muscle actin (αSMA) antibody (clone 1A4; Dako), anti‐FGFR3 antibody (clone B‐9; Santa Cruz), and anti‐FGFR4 antibody (clone A‐10; Santa Cruz). Immunohistochemical analyses were performed according to the manufacturer's instructions. Briefly, the slides were deparaffinized and heated for 10 min at 105°C in an autoclave with a citric acid solution (pH 6.0). The samples were incubated with primary antibodies for 24 h at 4°C after blocking endogenous peroxidase activity. Samples were incubated with biotinylated secondary antibodies for 1 h. The samples were treated with a streptavidin‐peroxidase reagent, and counterstained with Mayer's hematoxylin [19].

### 2.9 Preparation of Conditioned Medium (CM)

CM was collected from Nmeso and CAmeso cells. Cells were seeded in a 10 cm dish and cultured in 10% FBS/DMEM. After 24 h of incubation, the cells were washed thrice with PBS and incubated for 3 days in 4.5 mL of DMEM without FBS. The supernatant was stored as CM at −20°C until use [20].

### 2.10 Wound Healing Assay

Human colorectal tumor, line 116 (HCT116) cells were cultured in 96‐well plates (Essen Image Lock; Essen Instruments). Once the cells reached semiconfluence, a wound was generated in the monolayer using a 96‐well WoundMaker (Essen BioScience Inc., Ann Arbor, MI). HCT116 cells were cultured in CMs with 5% FBS. Scratched fields were imaged every 3 h and monitored with Incucyte ZOOM live‐cell imaging system and software version 2015A (Essen Instruments) [21].

### 2.11 Invasion Assay

In vitro invasiveness was measured using a two‐chamber Matrigel invasion assay with Chemotaxicell chambers (Millipore, Billerica, MA, USA) containing a 12 μm pore membrane filter coated with 50 μg of the Matrigel (upper chamber) in a 24‐well culture plate (lower chamber). A total of 5 × 10^4^ (OMUCR‐1) or 1 × 10^4^ (HCT116) cells per chamber were seeded into the upper chamber with 1% FBS DMEM, and 500 μL of CM from mesothelial cells or DMEM was added to the lower chamber as a control with 5% FBS. Following a 24 h incubation period at 37°C in 5% CO_2_, cells that invaded through the pores to the lower surface of the membrane were fixed and stained using Diff Quik (Sysmex, Kobe, Japan) [21].

### 2.12 Xenograft Models and In Vivo Treatments

OMUCR‐1 cells were injected subcutaneously (1 × 10^6^ cells) with or without CAmeso cells (1 × 10^6^ cells) in 6‐week‐old nude mice (female, *n* = 3/group). Tumors were harvested 6 weeks after subcutaneous injection. For therapeutic studies, nude mice (female, *n* = 10/group) were treated once daily with control vehicle or BGJ398 (Selleck, 10 mg/kg/dose, oral gavage) after implantation. Fifteen days after subcutaneous injection, tumors were harvested for staining.

### 2.13 RNA Sequencing and Computational Analysis

Harvested xenograft tumors were homogenized in TRIzol, whereas total RNA was extracted using the RNeasy Mini Kit according to the manufacturer's instructions. Bulk RNA‐seq analyses were performed in R (v4.3.2) and Python (v3.12) on the SHIROKANE high‐performance computing system at the Human Genome Center, The University of Tokyo (https://supcom.hgc.jp).

#### 2.13.1 Read Preprocessing and Mapping

Raw FASTQ files were adapter‐ and quality‐trimmed with Trim Galore (v0.6.10; Cutadapt wrapper). To ensure removal of residual adapters or low‐quality bases, trimming was iterated across three passes, and reads failing Trim Galore's default quality filters were discarded. A combined (concatenated) STAR index was built to distinguish human and mouse reads from xenografts, using the human GRCh38 primary assembly (annotation: GENCODE v38) and the mouse GRCm39 assembly (annotation: GENCODE vM32). Reads were aligned to this mixed reference with STAR (v2.7.10b). Mapping statistics, including overall and uniquely mapped read fractions, were obtained from STAR Log.final.out files and summarized in R.

#### 2.13.2 Species Disambiguation

Post‐alignment, reads uniquely mapped to either the human (GRCh38) or the mouse (GRCm39) genome were retained, whereas multi‐mapped or cross‐mapped reads were removed, generating human‐ and mouse‐only BAM/FASTQ sets for downstream analyses.

#### 2.13.3 Quantification and Normalization

Gene‐level raw counts were generated with featureCounts (Subread v2.0.5) using the corresponding GENCODE gene transfer formats. These raw integer counts were used for differential expression. For visualization and unsupervised analyses, transcripts per million (TPM) values were computed with RSEM (v1.3.3); genes with TPM = 0 across all samples were excluded from principal component analysis (PCA) and heatmaps.

#### 2.13.4 Downstream Analyses and Visualization

PCA was performed on log2(TPM + 1) matrices. Differentially expressed genes (DEGs) were identified with DESeq2 (Bioconductor; v1.40.2) using the raw count matrix; multiple‐testing correction used the Benjamini–Hochberg's procedure (significance thresholds specified in figure legends). Heatmaps and summary plots were generated in R using ggplot2, dplyr, and reshape2.

#### 2.13.5 Software and Reference Versions

Software and versions included Trim Galore v0.6.10; STAR v2.7.10b; Subread/featureCounts v2.0.5; RSEM v1.3.3; R v4.3.2; and Python v3.12. References genomes were human GRCh38 (GENCODE v38) and mouse GRCm39 (GENCODE vM32). Key R packages included DESeq2 (Bioconductor, v1.40.2), ggplot2 (v3.5.0), dplyr (v1.1.4), and reshape2 (v1.4.4).

### 2.14 Statistical Analysis

Data are expressed as mean ± SEM, and statistical significance was assessed using the unpaired Student's *t*‐test. Statistical significance was set at *p* < 0.05. Statistical analysis was performed using GraphPad Prism 10.

## 3 Results

### 3.1 Syngeneic Establishment of Rectal Cancer and Mesothelial Cell Lines From the Malignant Ascites of a Single Patient

**FIGURE 1:**
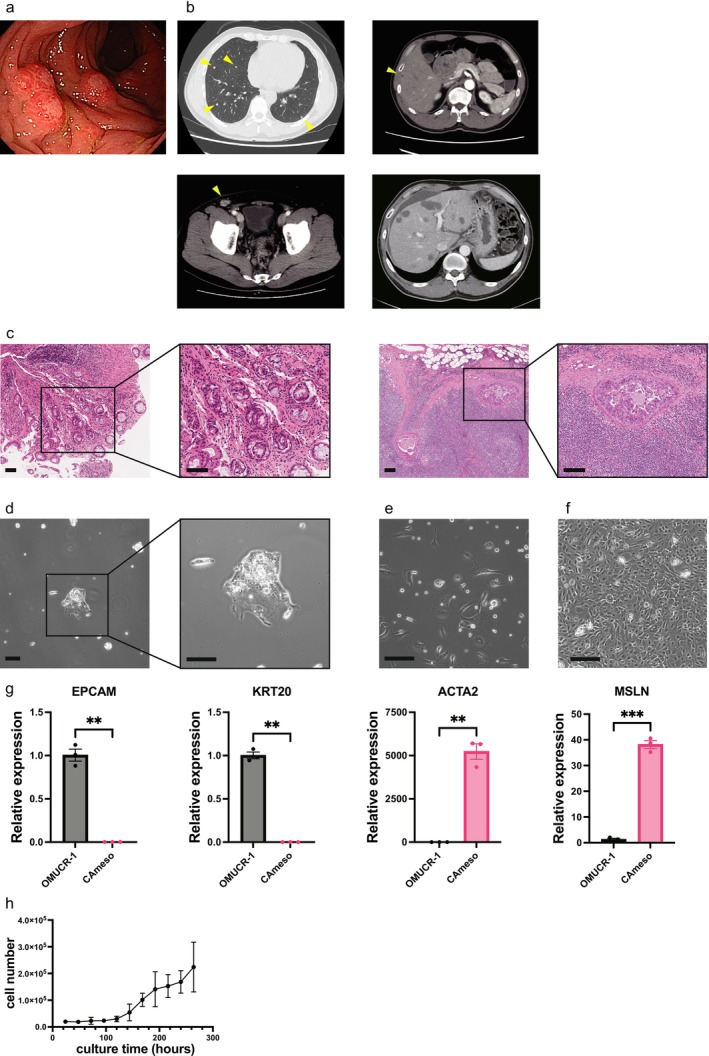
Clinicopathological features of the patient, along with the morphological and molecular profiling of OMUCR‐1 and CAmeso cells. (a) Macroscopic findings of colonoscopy. The tumor presented as a type 1 lesion with an elevated mucosa. (b) CT images. Thoracic CT reveals multiple lung metastases (arrowheads). Abdominal CT shows liver metastasis (arrowhead), ascites, and swelling of the right inguinal lymph node (arrowhead). (c) Histopathological findings of a primary tumor biopsy and a right inguinal lymph node. The tumor was diagnosed as a poorly differentiated adenocarcinoma with lymph node metastasis from rectal cancer. Scale bar, 100 μm. (d) Phase‐contrast photomicrographs of live OMUCR‐1 cells. Scale bar, 200 μm. Phase‐contrast photomicrograph of living CAmeso cells. Scale bar, 200 μm. Phase‐contrast photomicrography of living immortalized CAmeso cells. Scale bar, 200 μm. (g) Relative mRNA expression of *EPCAM*, *KRT20*, *ACTA2* and *MSLN* expression in CAmeso cells compared with OMUCR‐1 cells measured by qPCR. Data are presented as mean ± SEM **p < 0.01, ***p < 0.001. (h) Growth curve of OMUCR‐1 cells. CT, computed tomography; qPCR, quantitative polymerase chain reaction; SEM, standard error of the mean.

Total colonoscopy revealed that the primary rectal tumor was a macroscopic type 1 tumor (Figure 1a). Computed tomography imaging revealed multiple metastases in the lungs and liver. The enlarged right inguinal lymph nodes were biopsied to confirm metastatic rectal cancer. Malignant ascites that developed during the course of treatment are presented in Figure 1b. Histopathological findings of the primary tumor and inguinal lymph node metastasis revealed a poorly differentiated adenocarcinoma (Figure 1c). Biopsy of the rectal lesion showed small nests of atypical cells distributed in the stroma.

OMUCR‐1 was successfully established from the malignant ascites of a patient. Notably, mesothelial cells (CAmeso) were derived from the same patient, which provided a rare opportunity to study both tumor and stromal cells in a single clinical case. Phase‐contrast microscopy revealed that OMUCR‐1 cells grew predominantly as floating clusters, with some cells exhibiting partial adherence (Figure 1d). These cells were cultured for > 24 months and subjected to > 120 passages. In contrast, CAmeso cells displayed a fully adherent morphology (Figure 1e) and were successfully immortalized (Figure 1f). STR analysis confirmed that OMUCR‐1 cells were unique and not identical to any cell lines in the JCRB Cell Bank database. To molecularly confirm the identities of OMUCR‐1 and CAmeso, qPCR for epithelial and mesothelial‐mesenchymal lineage markers was performed. OMUCR‐1 cells showed robust expression of the epithelial markers EPCAM and KRT20, whereas CAmeso displayed minimal expression of these genes. In contrast, CAmeso exhibited markedly elevated expression of the mesothelial‐mesenchymal markers ACTA2 and MSLN, whereas OMUCR‐1 expressed these genes at very low levels (Figure 1g). These mutually exclusive expression patterns confirm that OMUCR‐1 and CAmeso are phenotypically distinct, ruling out cross‐contamination between the two cultures.

The doubling time of OMUCR‐1 was calculated to be 27.9 h (Figure 1h). Drug sensitivity assays revealed that OMUCR‐1 was responsive to standard CRC chemotherapeutics, including 5‐FU, oxaliplatin, and irinotecan, with the half‐maximal inhibitory concentration (IC_50_) values of 0.29, 3.59, and 0.53 μM, respectively (Figure S1a). Cancer panel sequencing analysis revealed mutations in the Kirsten rat sarcoma viral oncogene homolog (KRAS) and the tumor protein p53 (TP53). Mutations in KRAS (NM_004985: c.35G > A; p.Gly12Asp) and in TP53 (NM_000546: c.916C > T; p.Arg306*) were predicted to be pathogenic. No actionable variants of fusion genes or copy number alterations were detected (Table S2). These results were consistent with mutation testing of the patient's clinical specimens, which showed a KRAS codon 12 mutation, wild‐type BRAF, and microsatellite stability.

### 3.2 Tumorigenic Potential of OMUCR‐1 Cellsss

**FIGURE 2:**
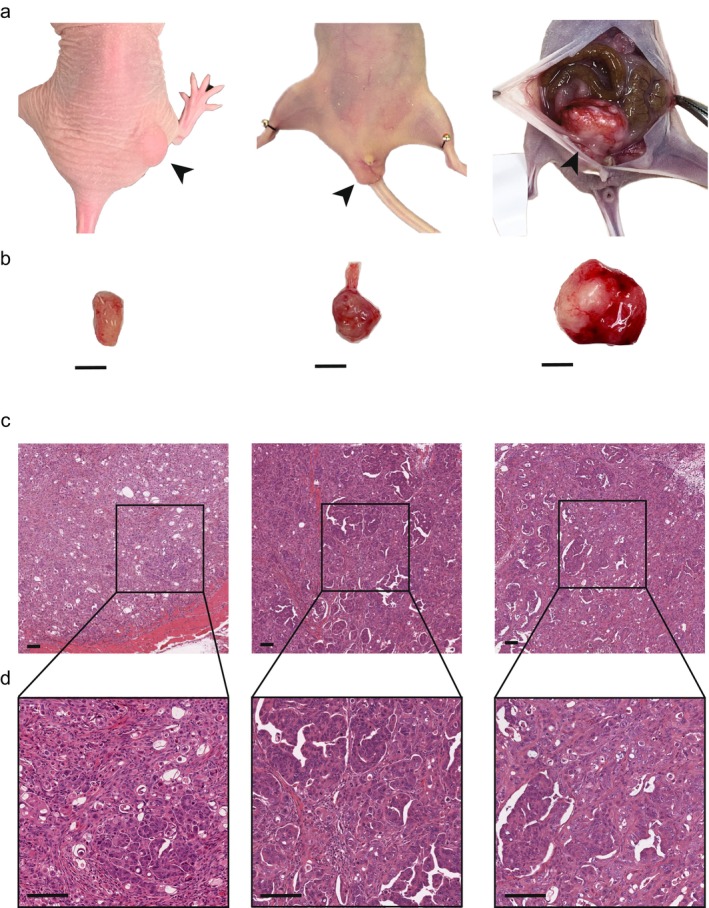
OMUCR‐1 cells exhibit tumorigenic potential in multiple in vivo models. (a, b) Gross appearance of tumors generated by subcutaneous, orthotopic, or intraperitoneal inoculation. Scale bar, 5 mm. (c, d) H&E staining of tumors. Scale bar, 100 μm. H&E, hematoxylin and eosin.

OMUCR‐1 cells exhibited strong tumorigenicity in multiple in vivo models. Subcutaneous inoculation of 1 × 10^6^ OMUCR‐1 cells into mice resulted in tumor formation in 100% of cases (12/12). Orthotopic implantation of 5 × 10^5^ OMUCR‐1 cells into the rectum led to tumor development in 58.3% of the cases (7/12). In addition, intraperitoneal injection led to tumor formation, highlighting the robust tumorigenic capacity of OMUCR‐1 cells (Figure 2a–c). These findings indicate that OMUCR‐1 cells possess strong tumorigenic potential, supporting their utility as a reliable in vivo experimental model.

### 3.3 Expression of FGFRs in OMUCR‐1 Cells

**FIGURE 3:**
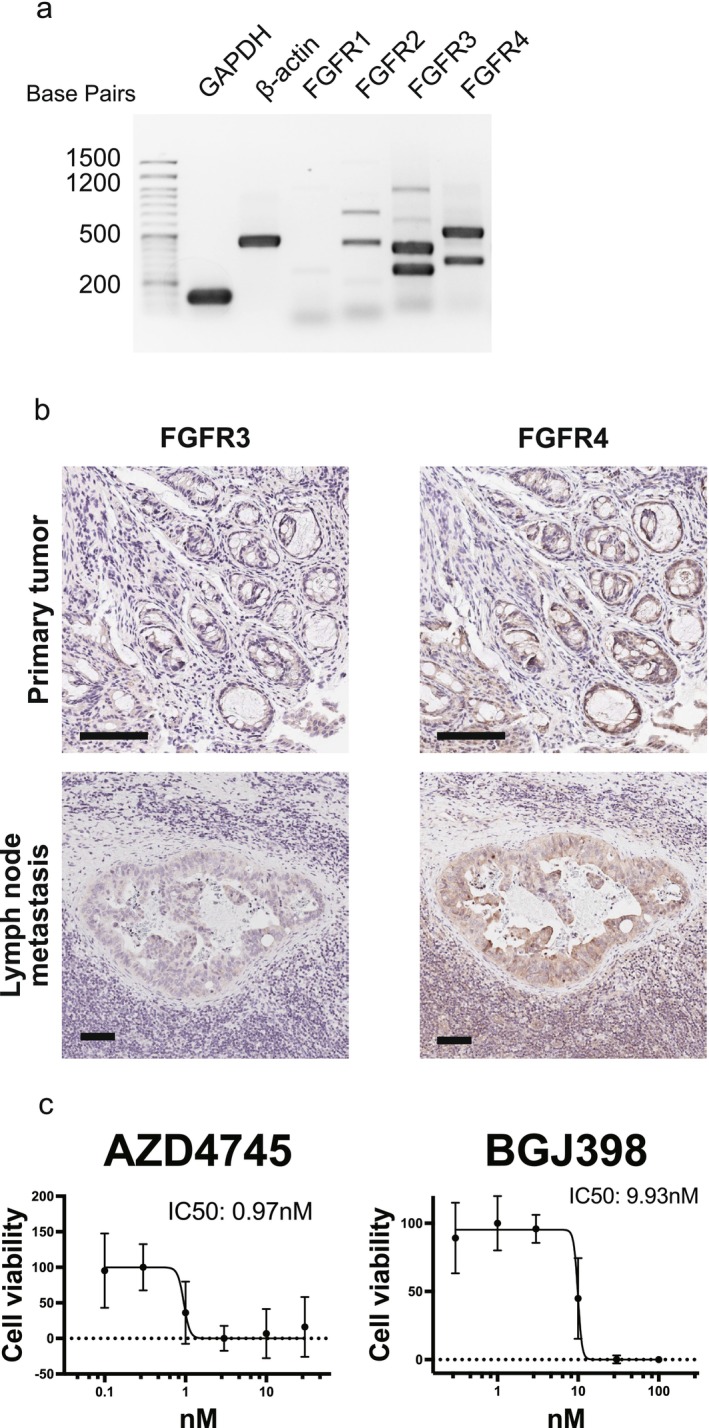
OMUCR‐1 cells express FGFR and are growth‐inhibited by FGFR inhibitors. (a) RT PCR analysis of FGFRs. (b) IHC for FGFR3 and FGFR4. Both the primary tumor biopsy and the inguinal lymph node metastasis show the expression of FGFR4 and FGFR3. Scale bar, 100 μm. (c) Effects of FGFR inhibitors (AZD4547 and BGJ398) on OMUCR‐1 cell proliferation. RT PCR, reverse transcription polymerase chain reaction; FGFR, fibroblast growth factor receptor; IHC, immunohistochemistry.

RT‐PCR analysis demonstrated that OMUCR‐1 cells express FGFR3 and FGFR4. Immunohistochemical analysis of the tumor showed strong FGFR4 expression and weak FGFR3 expression (Figure 3b). Treatment with the FGFR inhibitors, AZD4547 and BGJ398, reduced cell viability, with IC_50_ values of 0.97 and 9.93 nM, respectively (Figure 3c).

### 3.4 Effect of CAmeso on the Invasive Abilities of CRC Cells

**FIGURE 4:**
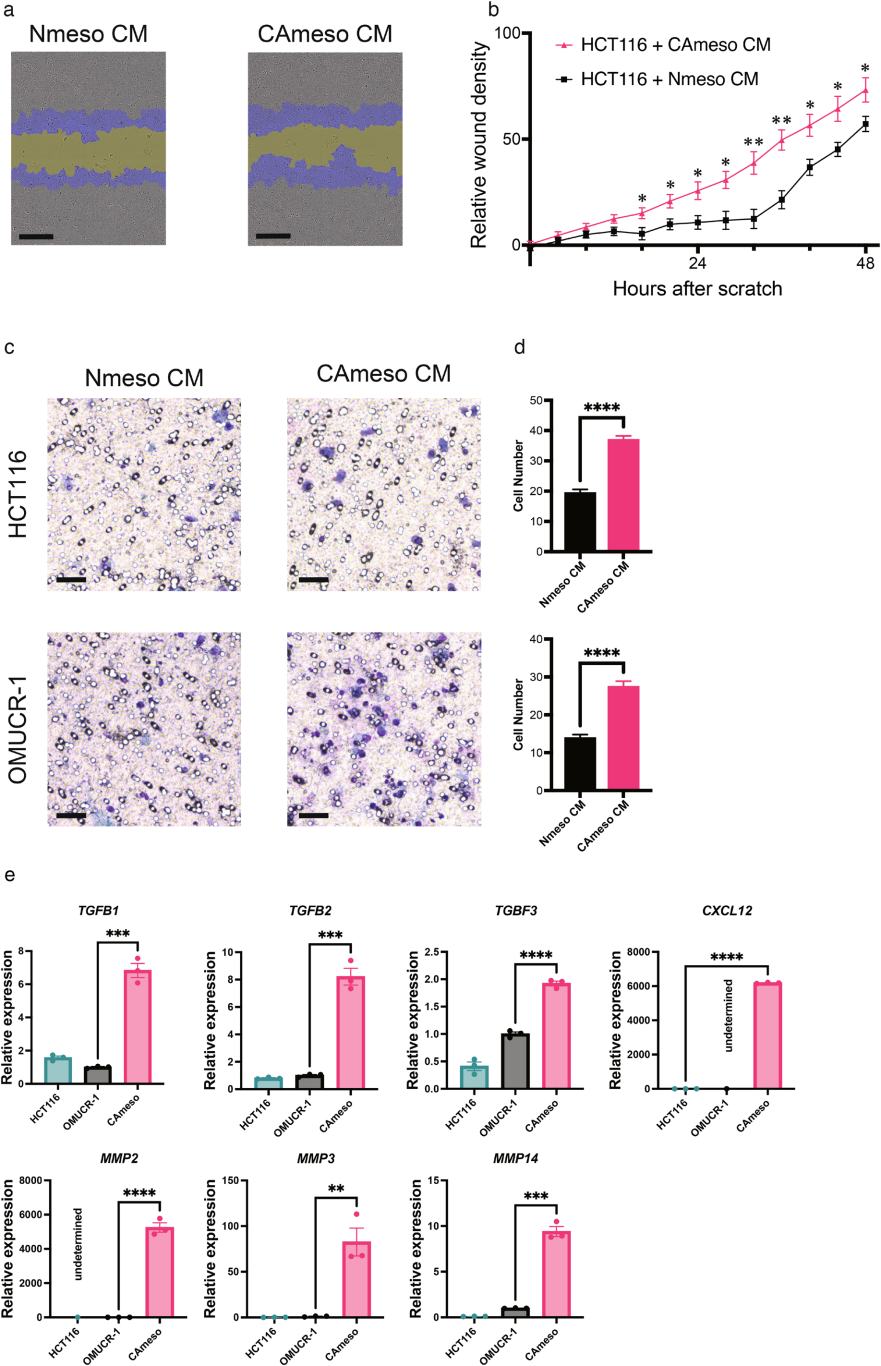
CAmeso‐conditioned medium promotes tumor cell migration and invasion, consistent with a pro‐invasive gene expression profile in CAmeso cells. (a) Representative images of the wound‐healing assay. Scale bar, 300 μm. (b) Effect of CAmeso conditioned medium on the migration of HCT116 cells. (c) Representative images of invading OMUCR‐1 and HCT116 cells. Scale bar, 100 μm. (d) Effect of CAmeso conditioned medium on OMUCR‐1 and HCT116 cell invasion. (e) Relative mRNA expression of *TGFB1*, *TGFB2*, *TGFB3*, *CXCL12*, *MMP2*, *MMP3*, and *MMP14* in CAmeso cells compared with OMUCR‐1 and HCT116 cells measured by qPCR. Data are presented as mean ± SEM *p < 0.05, **p < 0.01, ***p < 0.001, ****p < 0.0001. HCT116, human colorectal tumor, line 116; TGF, transforming growth factor; CXCL12, C‐X‐C motif chemokine ligand 12; MMP, matrix metalloproteinase; qPCR, quantitative polymerase chain reaction; SEM, standard error of the mean.

Figure 4a shows a representative phase‐contrast image of the wound‐healing assay conducted in this study. The images show the initial wound mask at 0 h (yellow) and the wound mask at 14 h (blue). Relative wound confluence (%) was calculated as 100 × (wound closure area at each time point [blue]/wound area at time zero [yellow]). The number of migrating HCT116 cells was significantly increased by CM from CAmeso compared with that from Nmeso (Figure 4b). Figure 4c shows a representative bright‐field image of OMUCR‐1 and HCT116 cells that invaded through a 12‐μm‐pore membrane filter. The number of invading OMUCR‐1 and HCT116 cells was significantly increased by CAmeso CM treatment (Figure 4d). The effect of CAmeso CM on cancer cell proliferation was evaluated; however, it did not significantly enhance cell growth (Figure S2a). To investigate the factors by which CAmeso‐derived CM promotes invasive capacity, the gene expression of growth factors, chemokines, and matrix metalloproteinases (MMPs) in mesothelial cells was examined. We found that transforming growth factor beta (TGF‐β), C‐X‐C motif chemokine ligand 12 (CXCL12), and various MMPs were elevated compared with cancer cells such as OMUCR‐1 and HCT116 (Figure 4e). These results suggest that CAmeso exerts tumor invasion‐promoting effects.

### 3.5 CAmeso Contributes to Tumor Growth In Vivo

**FIGURE 5:**
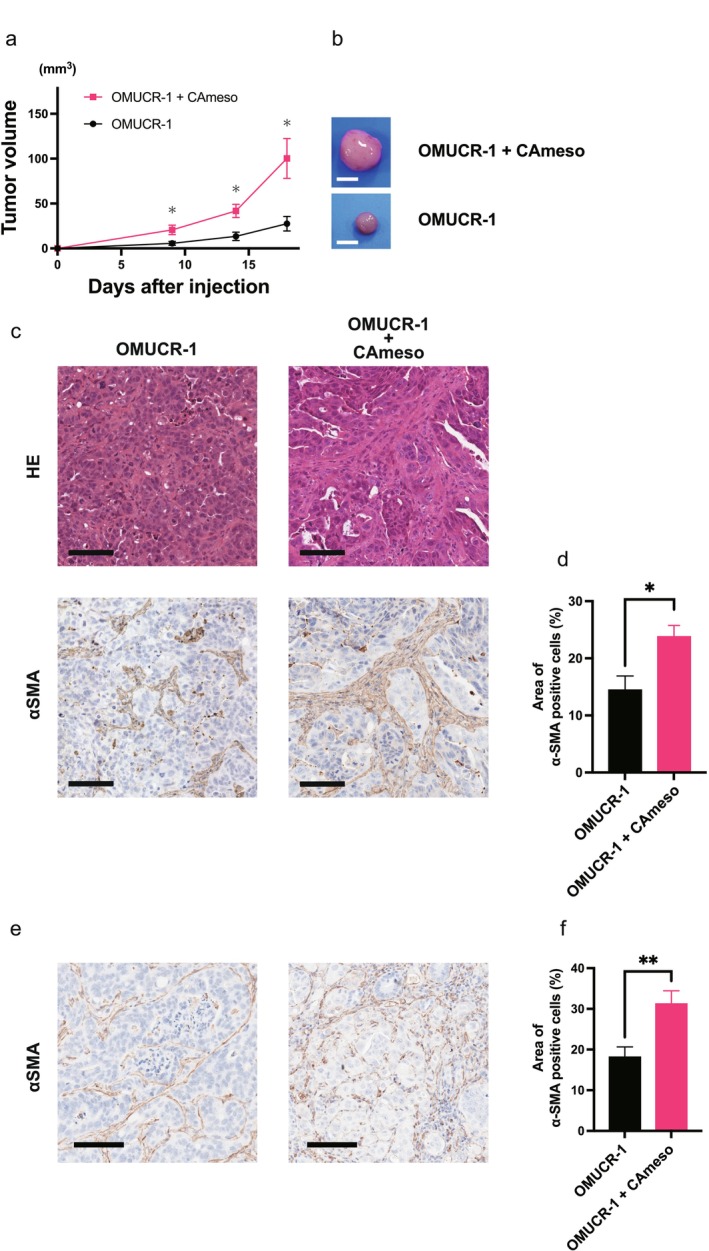
Co‐transplantation of OMUCR‐1 and CAmeso cells enhances tumor growth and increases stromal components. (a) Average tumor volume curve for subcutaneous inoculation of the OMUCR‐1 cells group and co‐inoculation with the CAmeso cells group. (b) Gross appearance of resected tumors. Scale bar, 5 mm. (c) Histological images of H&E staining and αSMA IHC of tumors from each group. Scale bar, 100 μm. (d) Quantification of the αSMA‐positive area rate. (e) Histological images of the αSMA IHC staining in orthotopic transplanted tumors in each group. Scale bar, 100 μm. (f) Quantification of the αSMA‐positive area rate. Data are presented as mean ± SEM *p < 0.05, **p < 0.01. H&E, hematoxylin and eosin; αSMA, α‐smooth muscle actin; IHC, immunohistochemistry; SEM, standard error of the mean.

In subcutaneous co‐transplantation models of nude mice, tumors formed by OMUCR‐1 cells with CAmeso were significantly larger than those formed by OMUCR‐1 cells alone (*p* < 0.05) (Figure 5a,b). Histological analysis demonstrated a significantly greater amount of αSMA‐positive stromal components (*p* = 0.042) in tumors co‐transplanted with CAmeso than in those formed by OMUCR‐1 alone (Figure 5c,d). Similarly, in orthotopic transplantation models, tumors formed by co‐transplantation exhibited significantly higher stromal content than those formed by OMUCR‐1 alone (*p* = 0.008) (Figure 5e,f). These results suggest that CAmeso contributes to tumor growth, promoting fibrotic remodeling within the tumor microenvironment.

### 3.6 Co‐Transplanted Tumors Were Suppressed by the FGFR Inhibitor

**FIGURE 6:**
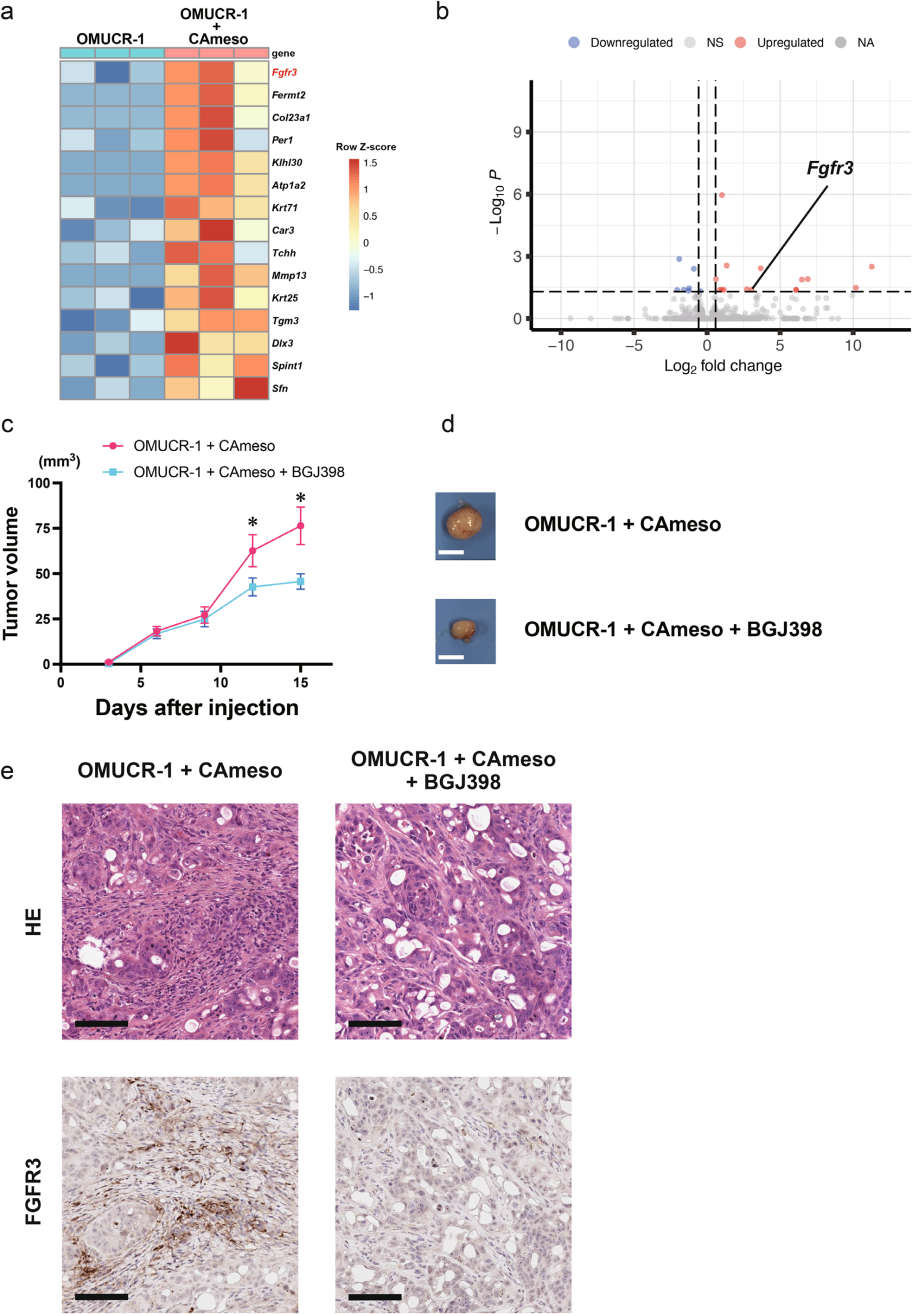
FGFR inhibition suppresses tumor growth and stromal activation in co‐transplanted tumors. (a) Heatmap of 15 murine genes upregulated in tumors transplanted with CAmeso relative to those without CAmeso. Colors indicate row‐wise Z‐scores of log2‐normalized counts from DESeq2's normTransform. Columns are individual samples; the top annotation denotes Condition (OMUCR‐1 vs. OMUCR‐1 + CAmeso). (b) Volcano plot of DEGs of murine genes. (c) Average tumor volume curve for the BGJ398‐treated and control groups. Data are presented as mean ± SEM *p < 0.05. (d) Gross appearance of resected tumors. Scale bar, 5 mm. (e) Histological images of H&E staining and FGFR3 IHC of tumors from each group. Scale bar, 100 μm. FGFR, fibroblast growth factor receptor; DEG, differentially expressed genes; SEM, standard error of the mean; H&E, hematoxylin and eosin; IHC, immunohistochemistry.

Total RNA extracted from tumors with or without the addition of CAmeso cells to OMUCR‐1 cells was analyzed by RNA sequencing. The number of DEGs identified was 11 for human genes and 22 for mouse genes. Among these, genes with higher expression in tumors with CAmeso were 1 human gene and 15 mouse genes. Focusing on mouse genes, the expression of *Fgfr3* was found to be elevated in tumors with CAmeso (Figure 6a,b). Tumors co‐transplanted with CAmeso exhibited a trend toward increased expression of invasion‐related genes, including TGF‐β and MMPs, which was consistent with the in vitro findings (Figure S3a). Treatment of tumors containing CAmeso cells with BGJ398 resulted in a reduction in tumor size (Figure 6c,d). Histological analysis demonstrated a reduction of FGFR3‐positive stromal components in tumors with FGFR inhibitor treatment compared with those without treatment (Figure 6e). Collectively, these findings indicate that the FGFR inhibitor not only exerts direct effects on tumor cells but also modulates tumor‐promoting stromal activity within the microenvironment.

## 4 Discussion

Peritoneal dissemination represents a severe and often terminal stage of CRC; however, experimental models replicating its underlying biology remain limited. In our study, we established a novel autologous paired system in which OMUCR‐1 and CAmeso were simultaneously derived from the malignant ascites of the same patient. This unique model provides a physiologically relevant platform for investigating tumor‐mesothelial crosstalk that drives peritoneal metastasis.

Mesothelial cells and mesothelial‐derived stromal populations are implicated in creating a permissive microenvironment for metastatic implantation [13, 22]. The importance of mesothelial cells and mesothelial‐derived populations has been noted in studies of peritoneal dissemination and the cancer microenvironment [23, 24]. Although widely used CRC cell lines such as HCT116, LoVo, and SW480 [25] advance the understanding of CRC biology, only a few cell lines are derived from patients with peritoneal dissemination. Notably, this is the first study describing the concurrent establishment of both CRC cells and CAmeso from the same clinical specimen. The distinct growth properties of OMUCR‐1 (suspension) and CAmeso (adherent) facilitated their separation, with STR profiling confirming OMUCR‐1 as a unique CRC cell line. qPCR‐based lineage marker analysis further validated the phenotypic identities of each line, demonstrating that OMUCR‐1 selectively expressed epithelial markers (EPCAM and KRT20), whereas CAmeso selectively expressed mesothelial‐mesenchymal markers (ACTA2 and MSLN). These results rule out cross‐contamination and confirm the distinct origins of the two cell populations.

Beyond phenotypic identity, molecular features relevant to therapeutic responsiveness were also explored. Conventional RT‐PCR demonstrated expression of multiple FGFRs in OMUCR‐1, and immunohistochemical staining confirmed FGFR3 and FGFR4 expression in the patient's primary tumor and lymph node metastasis. OMUCR‐1 exhibited partial sensitivity to AZD4547 and BGJ398, suggesting that FGFR signaling contributes to its growth. However, detailed mechanistic analyses were beyond the scope of this study, and further analyses are required to clarify whether FGFR3 or FGFR4 represent tumor‐cell‐intrinsic vulnerabilities.

In vivo, co‐transplantation of OMUCR‐1 with CAmeso increased tumor size and stromal expansion, including αSMA‐positive fibroblast‐like elements, in both subcutaneous and orthotopic models. RNA sequencing and IHC revealed upregulation of murine stromal *Fgfr3* in co‐transplanted tumors, suggesting activation of FGFR3‐related stromal pathways. Treatment with BGJ398 reduced tumor growth and decreased stromal FGFR3‐positive cells, indicating that FGFR inhibition may attenuate both tumor‐cell proliferation and stromal support. However, the therapeutic relevance of targeting stromal FGFR3 remains preliminary, warranting further investigation.

CAmeso exerted clear tumor‐promoting effects on CRC cells. Conditioned medium from CAmeso enhanced migration and invasion of OMUCR‐1 and HCT116 cells, consistent with previous studies showing that mesothelial‐mesenchymal transition promotes cancer cell invasiveness [13, 26, 27]. Kitayama et al. demonstrated that CD90^+^ mesothelial‐like cells present in peritoneal fluid enhance peritoneal metastasis in vivo, supporting the concept that free‐floating mesothelial progenitor cells contribute to metastatic niche formation within the peritoneal cavity [28]. Recent studies have emphasized the central role of Toll‐like receptor signaling in CRC pathogenesis, linking microbial dysbiosis to chronic inflammation, immune dysregulation, and tumor progression [29]. These insights support the relevance of investigating tumor‐stroma crosstalk in experimental systems, especially in anatomical contexts such as the peritoneal cavity, where inflammatory and stromal cues converge.

In our study, CAmeso CM enhanced oncogenic properties of OMUCR‐1 cells, indicating that soluble factors derived from CAmeso actively contribute to tumor‐promoting signaling. Our findings provide functional evidence for paracrine stromal regulation of CRC cells. In qPCR analysis of CAmeso and CRC cell lines, gene expression of *TGF‐β*, *CXCL12*, and *MMP*s, which promote cancer cell invasion, was elevated in CAmeso. Furthermore, in vivo co‐transplantation experiments of OMUCR‐1 and CAmeso cells into mice showed a trend toward increased expression of invasion‐related factors in co‐transplanted tumors. TGF‐β and CXCL12 promote tumor invasion [30, 31, 32]. MMPs facilitate tumor invasion through the degradation of extracellular matrix components, particularly type IV collagen within the basement membrane. Beyond matrix remodeling, MMPs promote tumor progression by activating growth factors such as TGF‐β and vascular endothelial growth factor, thereby enhancing proliferation, angiogenesis, and epithelial‐mesenchymal transition [33, 34]. MMPs are produced not only by cancer cells but also by stromal components, including cancer‐associated fibroblasts and macrophages, underscoring their central role in tumor‐stroma interactions [35].

In our study, a 3D in vitro culture model was not established. However, previous studies demonstrated that organotypic 3D co‐culture systems are valuable tools for recapitulating tumor‐stroma interactions and assessing cancer cell invasion and stromal activation [36]. Incorporating such 3D models is essential for future investigations to further characterize the functional properties of our established cells.

Recent pharmacological studies emphasized that CRC progression and treatment resistance are critically influenced by chronic inflammation and stromal‐immune interactions within the tumor microenvironment [37]. This highlights the need for experimental models that capture paracrine signaling between tumor cells and stromal compartments, particularly in advanced disease contexts such as peritoneal dissemination.

This study has some limitations. First, CAmeso was immortalized using SV40 large T antigen and hTERT, which may modify certain biological behaviors. Second, both cell lines were derived from a single patient, limiting generalizability. Third, the co‐transplantation model used equal cell numbers, which may have partially influenced tumor growth dynamics. Despite these limitations, our lineage marker analysis and functional assays strongly support the biological interaction between CRC cells and mesothelial cells.

In conclusion, our study presents a unique autologous experimental model that enables direct investigation of tumor‐mesothelial interactions within the peritoneal microenvironment. This system offers a valuable platform for exploring the stromal mechanisms that drive peritoneal dissemination. Although our findings highlight stromal FGFR3 as a potential microenvironmental vulnerability, further studies are needed to define its therapeutic significance. Continued investigation using this model may yield important insights into the biology of peritoneal metastasis and inform the development of new treatment strategies for advanced CRC.

## Author Contributions

**Yasuhiro Fukui** and **Hiroaki Kasashima:** conceptualization. **Yasuhiro Fukui**, **Hiroaki Kasashima**, **Yu Muta**, **Yuki Nakanishi** and **Masakazu Yashiro:** methodology. **Yasuhiro Fukui**, **Zizhou Wang**, **Iguru Omori**, **Yukina Kusunoki**, **Kanae Echizen** and **Yuki Nakanishi:** formal analysis and investigation. **Yasuhiro Fukui:** writing – original draft preparation. **Hiroaki Kasashima**, **Yuki Seki**, **Kenji Kuroda**, **Yuichiro Miki**, **Mami Yoshii**, **Tatsunari Fukuoka**, **Tatsuro Tamura**, **Masatsune Shibutani**, **Takahiro Toyokawa**, **Yu Muta**, **Yuki Nakanishi**, **Naoko Ohtani**, **Masakazu Yashiro** and **Kiyoshi Maeda:** writing – review and editing. **Hiroaki Kasashima:** funding acquisition. **Hiroaki Kasashima** and **Kiyoshi Maeda:** resources. **Hiroaki Kasashima**, **Yu Muta**, **Yuki Nakanishi**, **Naoko Ohtani**, **Masakazu Yashiro** and **Kiyoshi Maeda:** supervision. All authors have read and agreed to the published version of the manuscript.

## Funding

The study was supported in part by Grants‐in‐Aid for Scientific Research from the Japan Society for the Promotion of Science (JSPS KAKENHI, Grant nos. 21K16428 and 23K15480), the foundations of Takeda Science (H. K.), the SGH Cancer Research Grants (H. K.), the Kobayashi Foundation for Cancer Research (H. K.), the Yasuda Medical Foundation (H. K.), the Osaka Community Foundation (H. K.), the Uehara Memorial Foundation, (H. K.), Kanae Foundation for the Promotion of Medical Science (H. K.), the Mochida Memorial Foundation for Medical, Pharmaceutical Research (H. K.), the Japan Agency for Medical Research and Development (AMED, S250264) (H.K.), the Medical Research Encouragement Prize of The Japan Medical Association (H. K.), Research Grant of the Princess Takamatsu Cancer Research Fund (H. K.), and The Ichiro Kanehara Foundation (H. K.).

## Ethics Statement

All animal experiments were performed in compliance with the guidelines of the Osaka Metropolitan University Ethical Committee.

## Consent

Informed consent was obtained from the patients.

## Conflicts of Interest

The authors declare no conflicts of interest.

## Supporting information


**Figure S1:** (a) Effects of 5‐FU, oxaliplatin, and irinotecan on OMUCR‐1 cell proliferation.
Data are presented as mean ± SEM. 5‐FU, 5‐fuluoro uracil; SEM, standard error of the mean.
**Figure S2:** (a) Effect of CAmeso CM on OMUCR‐1 and HCT116 cell proliferation.
Data are presented as mean ± SEM. CM, conditioned medium; SEM, standard error of the mean.
**Figure S3:** (a) Mouse gene expressions in tumors created by subcutaneous transplantation. Data are presented as mean ± SEM *p < 0.05, **p < 0.01. SEM, standard error of the mean.
**Table S1:** Primer used.
**Table S2:** Results of cancer panel analysis.

## Data Availability

The datasets generated and analyzed during the current study are not publicly available because of patient privacy and institutional restrictions; however, the datasets are available from the corresponding author upon reasonable request. RNA‐sequencing data contain patient‐derived sequences that cannot be fully anonymized; therefore, deposition in a public repository is not permitted under the terms of our ethical approval.
