# Supplementary material for: Simultaneous Establishment of Autologous Colorectal Cancer and Mesothelial Stromal Cell Lines from Malignant Ascites Reveals a Mesothelial‐Stromal FGFR3 Axis as a Potential Vulnerability in Peritoneal Metastasis
Source: Cancer Med. 2026 Apr 24;15(5):e71804. doi: 10.1002/cam4.71804 (PMC13109080; doi:10.1002/cam4.71804)
Supplement: Supplementary file 1 — Figure S1: (a) Effects of 5‐FU, oxaliplatin, and irinotecan on OMUCR‐1 cell proliferation. Data are presented as mean ± SEM. 5‐FU, 5‐fuluoro uracil; SEM, standard error of the mean. Figure S2: (a) Effect of CAmeso CM on OMUCR‐1 and HCT116 cell proliferation. Data are presented as mean ± SEM. CM, conditioned medium; SEM, standard error of the mean. Figure S3: (a) Mouse gene expressions in tumors created by subcutaneous transplantation. Data are presented as mean ± SEM *p < 0.05, **p < 0.01. SEM, standard error of the mean. Table S1: Primer used. Table S2: Results of cancer panel analysis. [file CAM4-15-e71804-s001.zip › 3_UL_supplementary_figure1.pdf]

Supplementary Figure 1

a

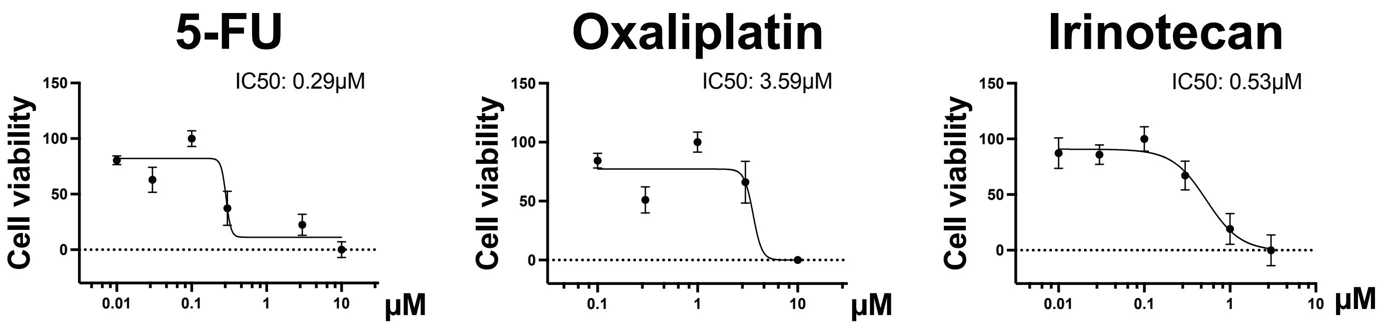

Supplementary Figure 1a:

Effects of 5-FU, oxaliplatin, and irinotecan on OMUCR-1 cell proliferation.

Data are presented as mean ± SEM.

Abbreviations: 5-FU, 5-fuluoro uracil; SEM, standard error of the mean
