# Supplementary material for: Simultaneous Establishment of Autologous Colorectal Cancer and Mesothelial Stromal Cell Lines from Malignant Ascites Reveals a Mesothelial‐Stromal FGFR3 Axis as a Potential Vulnerability in Peritoneal Metastasis
Source: Cancer Med. 2026 Apr 24;15(5):e71804. doi: 10.1002/cam4.71804 (PMC13109080; doi:10.1002/cam4.71804)
Supplement: Supplementary file 1 — Figure S1: (a) Effects of 5‐FU, oxaliplatin, and irinotecan on OMUCR‐1 cell proliferation. Data are presented as mean ± SEM. 5‐FU, 5‐fuluoro uracil; SEM, standard error of the mean. Figure S2: (a) Effect of CAmeso CM on OMUCR‐1 and HCT116 cell proliferation. Data are presented as mean ± SEM. CM, conditioned medium; SEM, standard error of the mean. Figure S3: (a) Mouse gene expressions in tumors created by subcutaneous transplantation. Data are presented as mean ± SEM *p < 0.05, **p < 0.01. SEM, standard error of the mean. Table S1: Primer used. Table S2: Results of cancer panel analysis. [file CAM4-15-e71804-s001.zip › 3_UL_SupTable1.pdf]

Supplymentary Table1
Primers used

**Gene symbol**	**Forward**	**Reverse**
EPCAM	CTGCTCTGAGCGAGTGAGAA	TCCTTCTGAAGTGCAGTCCG
KRT20	AGCTGCGAAGTCAGATTAAGGA	GAAGTCCTCAGCAGCCAGTT
ACTA2	TATCCCCGGGACTAAGACGG	AGAGCCATTGTCACACACCA
MSLN	CAGAGGAGGCTCAGAGAGCTA	GGTCGAGCCGTTGGCAAG
TGFB1	GGAAATTGAGGGCTTTCGCC	CCGGTAGTGAACCCGTTGAT
TGFB2	GCGACGAAGAGTACTACGCC	CGGGATGGCATTTTCGGAG
TGFB3	ATTCCGAGCAGAATTCCGGG	CTGGCCGAAGGATCTGGAAG
CXCL12	GCCCTTCAGATTGTAGCCCG	TGGCTGTTGTGCTTACTTGTT
MMP2	ACAAAGAGTTGGCAGTGCAATA	TCTGGGGCAGTCCAAAGAAC
MMP3	CACAGACCTGACTCGGTTCC	GAGTCAGGGGGAGGTCCATA
MMP14	CGGAGAATTTTGTGCTGCCC	AACCCTGACTCACCCCCATA
