# Supplementary material for: Simultaneous Establishment of Autologous Colorectal Cancer and Mesothelial Stromal Cell Lines from Malignant Ascites Reveals a Mesothelial‐Stromal FGFR3 Axis as a Potential Vulnerability in Peritoneal Metastasis
Source: Cancer Med. 2026 Apr 24;15(5):e71804. doi: 10.1002/cam4.71804 (PMC13109080; doi:10.1002/cam4.71804)
Supplement: Supplementary file 1 — Figure S1: (a) Effects of 5‐FU, oxaliplatin, and irinotecan on OMUCR‐1 cell proliferation. Data are presented as mean ± SEM. 5‐FU, 5‐fuluoro uracil; SEM, standard error of the mean. Figure S2: (a) Effect of CAmeso CM on OMUCR‐1 and HCT116 cell proliferation. Data are presented as mean ± SEM. CM, conditioned medium; SEM, standard error of the mean. Figure S3: (a) Mouse gene expressions in tumors created by subcutaneous transplantation. Data are presented as mean ± SEM *p < 0.05, **p < 0.01. SEM, standard error of the mean. Table S1: Primer used. Table S2: Results of cancer panel analysis. [file CAM4-15-e71804-s001.zip › 3_UL_SupTable2.pdf]

Supplymentary Table2
Results of Cancer Panel Analysis

SNV/INDEL

Gene	Transcript ID (exon no.)	DNA Change Protein Change	AF (%) (Alt/Total)	Exonic Effect	Clinical Significance
KRAS	NM_004985 (2/5)	c.35G>A p.Gly12Asp	99.7 (875/878)	missense variant	Pathogenic
TP53	NM_000546 (8/11)	c.916C>T p.Arg306*	99.4 (505/508)	stop gained	Pathogenic

Fusion Gene: No Variant

Copy Number Alteration (CNA)

Gene	Type	Locus	Copy ratio (log2)	Copy Number
MYC	GAIN	8q24.21	1.55214	6
